# Receptor Interacting Protein Kinases 1/3: The Potential Therapeutic Target for Cardiovascular Inflammatory Diseases

**DOI:** 10.3389/fphar.2021.762334

**Published:** 2021-11-18

**Authors:** Yiming Leng, Ying Zhang, Xinyu Li, Zeyu Wang, Quan Zhuang, Yao Lu

**Affiliations:** ^1^ Clinical Research Center of the 3rd Xiangya Hospital, Central South University, Changsha, China; ^2^ Transplantation Center of the 3rd Xiangya Hospital, Central South University, Changsha, China; ^3^ Xiangya School of Medicine, Central South University, Changsha, China; ^4^ Research Center of National Health Ministry on Transplantation Medicine, Changsha, China

**Keywords:** RIPK1, RIPK3, cardiovascular diseases, necroptosis, therapeutic

## Abstract

The receptor interacting protein kinases 1/3 (RIPK1/3) have emerged as the key mediators in cell death pathways and inflammatory signaling, whose ubiquitination, phosphorylation, and inhibition could regulate the necroptosis and apoptosis effectually. Recently, more and more studies show great interest in the mechanisms and the regulator of RIPK1/3-mediated inflammatory response and in the physiopathogenesis of cardiovascular diseases. The crosstalk of autophagy and necroptosis in cardiomyocyte death is a nonnegligible conversation of cell death. We elaborated on RIPK1/3-mediated necroptosis, pathways involved, the latest regulatory molecules and therapeutic targets in terms of ischemia reperfusion, myocardial remodeling, myocarditis, atherosclerosis, abdominal aortic aneurysm, and cardiovascular transplantation, etc.

## Introduction

Cardiovascular disease is the major reason of global death, especially in North America, Europe, and Asia (Braunwald, 1997; Breslow, 1997). Classical anti-hypertension and hypolipidemic therapies are usually used for coronary artery disease, atherosclerosis, and heart failure. It has been revealed that certain circulating immune cells could directly induce endothelia injury, or indirectly activate endothelia to express effector molecules to aggravate deterioration in atherosclerosis (Eriksson et al., 2001; Skalen et al., 2002). Recruited by activated/injured endothelia, circulating monocytes could migrate into atherosclerotic plaque and differentiate into macrophages to release inflammatory cytokines and up-regulate pattern-recognition receptors to accelerate the development of atherosclerosis (Peiser et al., 2002). Recent studies also indicated that cardiac hypertrophy, fibrosis, and remodeling could be regulated by several kinds of immune cells, including dendritic cell (Anzai et al., 2012; Choo et al., 2017), T cell (Hofmann et al., 2012; Weirather et al., 2014), and B cell (Guzik et al., 2007), etc.

The receptor interacting protein kinases (RIPK), especially RIPK1 and RIPK3, are key mediators in cell death and inflammation. In RIPK family, 7 members share a homologous kinase domain which have diverse functions (Zhang et al., 2010). The incipiently family member RIPK1 contains a death domain (DD) which can identify and bond with other DD-containing proteins, such as tumor necrosis factor receptor 1 (TNFR1), TNF-related apoptosis-inducing ligand (TRAIL), and CD95 (Fas) (Micheau and Tschopp, 2003). RIPK3 contains N-terminal kinase domain like other members and an intermediary domain (ID) containing RIP homotypic interaction motif (RHIM) on C-terminus, which can interact with RIPK1 (Sun et al., 1999). The combination of RIPK1 and RIPK3 via RHIM is essential to initiate the necroptosis signaling pathway. RIPK1-RIPK3 interaction in inflammatory and immunological response has attracted much attention in recent years. By mutating RHIM of RIPK1 in mice, Z-DNA-binding protein 1 (ZBP-1) could trigger necroptosis and induce perinatal lethality, skin inflammation, and colitis (Jiao et al., 2020). However, RIPK1 could prevent ZBP-1 activating RIPK3 autophosphorylation on Thr231 and Ser232. Potential mechanism may be closely connected to competitive binding with RIPK3 though RHIM (Newton et al., 2016).

The structure of RIPK1 and whether it is in the kinase active conformation dictate its function. When ubiquitinated, RIPK1’s conformation is enclosed, so it is conducive to form a platform which facilitates congregation of multiprotein complexes resulting in nuclear factor κB (NF-κB) and mitogen-activated protein kinase (MAPK) activation (Kelliher et al., 1998). In its kinase-active form, RIPK1 recruits RIPK3 to its RHIM domain and initiates cell death pathways. RIPK1 is constitutively expressed in most tissues, and it can be stimulated by stress signals, DR ligands, or T cell activation (Stanger et al., 1995). When caspase-8 is inhibited or absent, TNF can trigger alternative cell death of necroptosis, which is regulated in execution, but necrotic in morphology (He et al., 2009). These signaling events can occur downstream of various DRs (TNF, Fas, etc.) with some variations (Newton, 2015).

In this review, we discuss the latest progress in this fast-moving field, with a focus on the signaling pathways, and the physiological and pathological implications of RIPK1/3 mediated necroptosis, and the latent therapeutic targets in diverse cardiovascular diseases.

## Apoptosis and Necroptosis Signaling Pathway

TNF and its superfamily members such as factor-related apoptosis ligand (FasL) and TRAIL are potent inducers of cytokines and inflammation which can also lead the caspase activation, DNA fragmentation, chromatin condensation, and ultimately the progress of extrinsic apoptosis (Guicciardi et al., 2013). The binding of TNF and TNFR1 on the surface of the cell leads to the recruitment of RIPK1 and other adaptor proteins, ubiquitin ligases, kinases to form the Complex I. RIPK1 can bind directly to the death domain (DD) in TNFR1 or the DD-containing adaptor TRADD (Pobezinskaya et al., 2008), meanwhile through the TNFR-associated factors, such as TRAF2 and TRAF5 (which are adaptor proteins), the E3 ubiquitin ligases cellular inhibitors of apoptosis (cIAP1/2) and linear ubiquitin chain assembly complex (LUBAC) are recruited to the complex (Gerlach et al., 2011). The ubiquitin ligases complex creates a scaffold for the recruitment of kinase complexes composed of transforming growth factor-b-activated kinase 1 and TAK1-binding proteins 2 and 3 (TAK1/TAB2/TAB3) (Kanayama et al., 2004). The pivotal function of TAK1 in regulating necroptotic myocyte death, myocardial remodeling, and heart failure propensity was initially described by Li et al. (2014). To verify the particular function of TAK1, cardiac-specific ablation of TAK1 mice was generated for apoptosis and necroptosis spontaneously leading to myocardial remodeling. This phenomenon could be rescued by genetic deletion of TNFR1. TAK1 as a nodal regulator of TNFR1-mediated necroptosis was unraveled whereafter. With activation of the downstream IKK-NFκB pathway, TAK1 was bound with RIPK1 through promotion of TNFR1 ligation. When activity of TAK1 was compromised, RIPK1 dissociated from TAK1 and switched its interacting partners to bind caspase 8 and FADD, inducing necroptosis mediated by RIPK1-FADD-caspase 8 and the RIPK1-RIPK3 complexes (Guo et al., 2017). TRAF2 is also a bridging regulator of necroptotic cell death which shows cardioprotective function. Ablation of TRADD could block necroptosis and abrogate the RIPK1-RIPK3 necrosome formation; activation of TAK1 could inhibit the necrosome formation by inhibiting RIPK1-RIPK3-FADD interaction. These results identified TRADD as an upstream regulator and TAK1 as a downstream effector in regulation of RIPK1-RIPK3-MLKL necroptotic signaling by TRAF2 (Guo et al., 2017).

**FIGURE 1:**
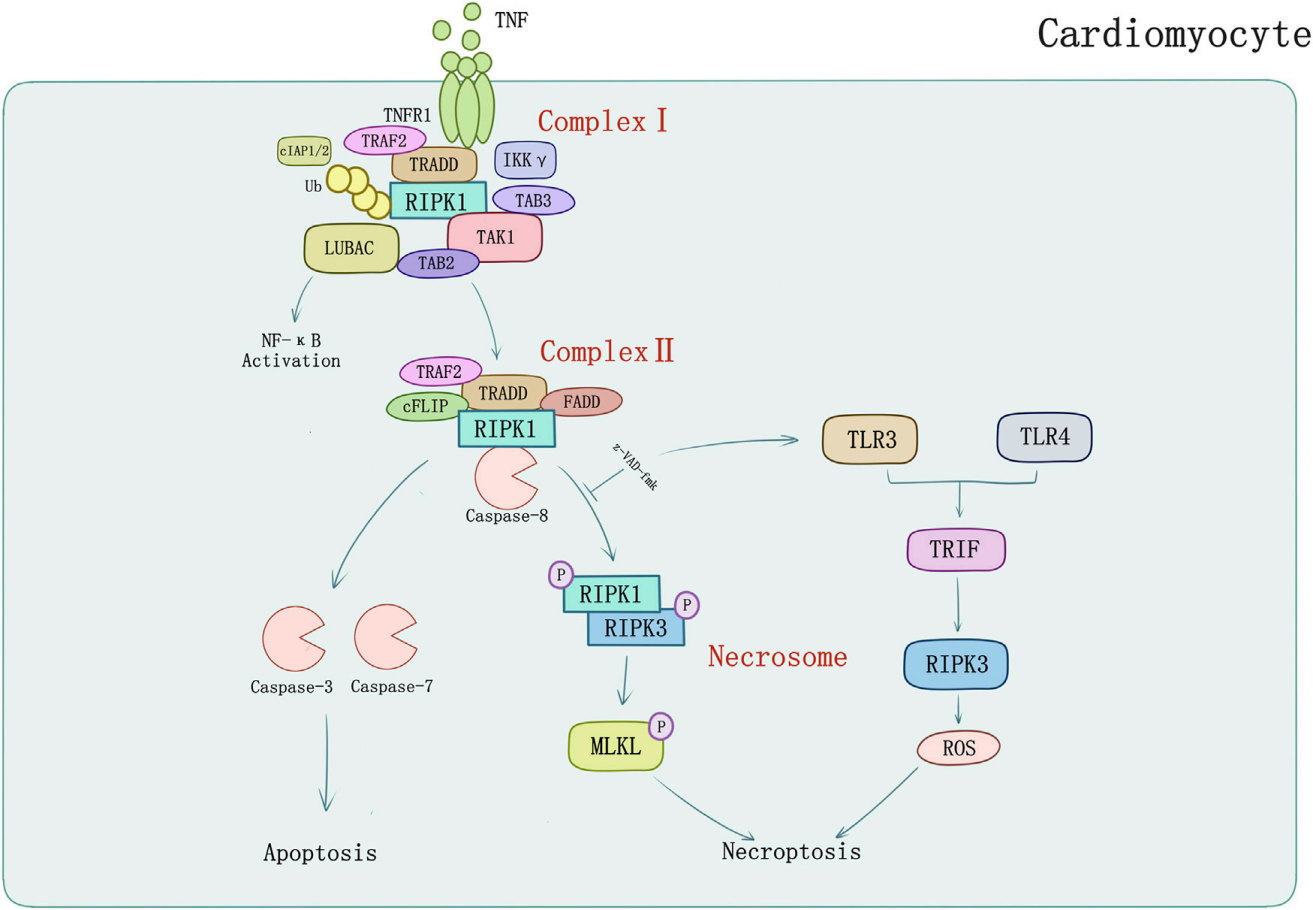
TNFR1-mediated apoptosis and necroptosis in cardiomyocyte. TNF-α binds with TNFR1 to transmit inflammatory signal into cells, and then induces formation of complex I consisting of TRADD, TRAF2, RIPK1, cIAPs, LUBAC, etc. Activated complex II induces caspase-dependent apoptosis which could be inhibited by pan-caspase inhibitor z-VAD-fmk. Z-VAD-fmk could also induce TLR3/TLR4-mediated signaling pathways and TRIF which induces RIPK3-mediated necroptosis through ROS.

Linear ubiquitin linkages on RIPK1 are important for recruiting NEMO/IKKγ which may inhibit necroptosis as well as apoptosis by binding to ubiquitinated RIPK1 to restrain RIPK1 from engaging the necroptotic death pathway (O’donnell et al., 2012; Shan et al., 2018). Further transformation from Complex I to cytoplasmic complex (Complex II) is required to execute apoptosis or necroptosis depending on the inhibit of cellular caspase-8/FLICE-like inhibitory protein (cFLIP) (Dillon et al., 2012). cFLIP can form heterodimers with caspase-8 in Complex II. In contrast with Complex I, the degradation of cIAPs or LUBAC, and the deubiquitylation of RIPK1 by cylindromatosis (CYLD) induce the formation of Complex II (Gerlach et al., 2011; Moquin et al., 2013) which has the same core components with ripoptosome (RIPK1/FADD/caspase-8). But ripoptosome forms independently of TNFR1 indicates that it cannot constitute Complex II (Feoktistova et al., 2011). Intrinsic apoptosis is triggered by apoptogenic proteins released by mitochondria which interrupts IAPs inhibition of caspases (Yang and Du, 2004) or activates caspase-9 (Li et al., 1997). The apoptosis promoted by Complex II requires the RIPK1, FADD, and caspase-8/cFLIP which lead the cascade of caspases (including caspase 8, 3, and 7) activation to perform apoptosis (Li et al., 1997; Micheau and Tschopp, 2003; Petersen et al., 2007). However, in situations where caspase inhibitors (e.g., z-VAD-fmk) block the activity of caspase-8 (and caspase-8/cFLIP heterodimers), the complex matures into necrosome which contains RIPK3 and MLKL (Holler et al., 2000; Degterev et al., 2005; Dillon et al., 2012). RIPK1 could auto-phosphorylate at Ser166 (Li et al., 2012) and interact with RIPK3 or other RHIM-containing proteins (TRIF for example) and induce downstream signaling pathways activation. RIPK1 binding to RIPK3 is a vital procedure to form necrosome which in turn activates RIPK3 phosphorylation at Ser 227 anthropogenically, or at Thr231 and Ser232 in murine (Li et al., 2012). RIPK3 can be directly activated by TLR3/TLR4-mediated signaling pathways and TRIF which induces RIPK3-mediated necroptosis through ROS without regulation of RIPK1 (He et al., 2011; Mocarski et al., 2011) (Figure 1). RIPK3 activation phosphorylates the pseudokinase MLKL (Sun et al., 2012). MLKL contains an N-terminal effector domain which is kept inactive by its C-terminal region folding in normal condition (Holler et al., 2000; Degterev et al., 2005). Bound with phosphorylated RIPK3, MLKL is phosphorylated thereupon and induces a conformational switch which exposes the N-terminal four-helix bundle of MLKL (Murphy et al., 2013). Oligomerization and translocation to plasma membrane of MLKL is induced, therefore, causing membrane rupture and cell death of which the explicit mechanisms require to be defined (Murphy et al., 2013; Cai et al., 2014; Hildebrand et al., 2014).

## RIPK1/3 in Cardiac Diseases

### The Crosstalk Between Necroptosis and Autophagy in Myocardial Death

**FIGURE 2:**
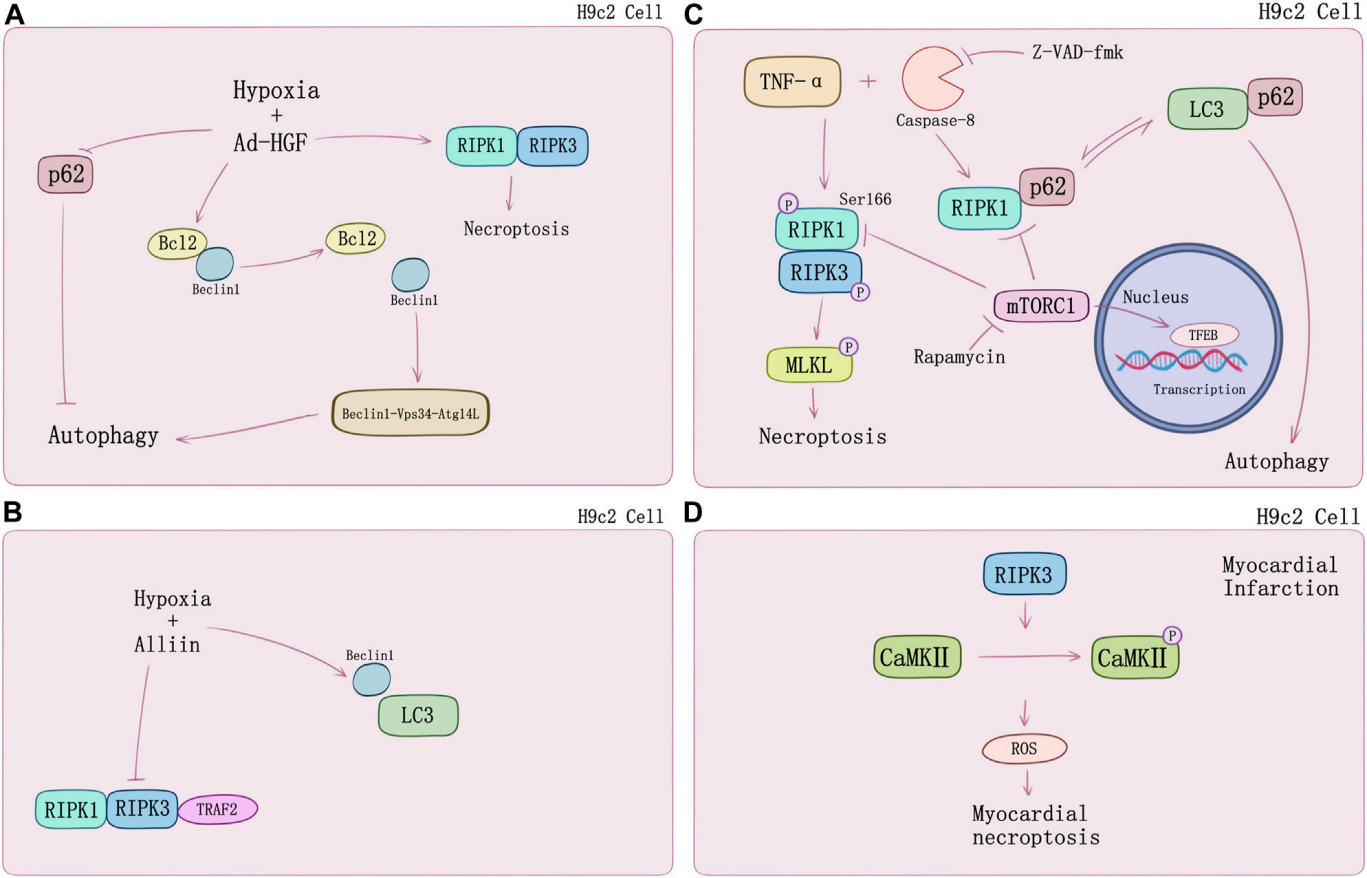
The crosstalk between necroptosis and autophagy in H9c2 cells. **(A)** HGF promotes autophagy and necroptosis in H9c2 cells under hypoxia by decreasing the binding of Bcl2 to Beclin1 and p62. **(B)** Alliin decreases hypoxia-induced necroptosis in H9c2 cells and promotes autophagy by increasing the levels of Beclin1 and LC3. **(C)** TNF-α/zVAD treatment induces necroptosis and switches RIP1-p62 binding to p62-LC3 notably causing suppression of autophagic flux. mTOC1 inhibition by rapamycin would suppress TNF-α/zVAD-mediated activation of IKKα/β and attenuates RIPK1/RIPK3-mediated necroptosis by suppressing RIP1-Ser166 phosphorylation. Rapamycin can induce TFEB nuclear translocation and promote autophagy in a TFEB-dependent manner. **(D)** RIP3-induced CaMKII phosphorylation and the release of ROS.

Necroptosis causes cardiomyocyte death in multiple signaling pathways. Luedde et al. (2014) first observed up-regulation of RIPK3 expression in ischemic mouse model. Following discovery argued that RIPK1-RIPK3-mediated necroptosis may play a special role in cardiomyocytes subsistence and death. Inhibiting HMGB1 expression via dexmedetomidine treatment could suppress hypoxia/reoxygenation (H/R)-induced necroptosis (Chen et al., 2019). However, the underlying mechanisms of myocardial death regulated by necroptosis still require thorough understanding. A growing number of new research suggests that crosstalk with autophagy may provide novel perspectives. Autophagy is an evolutionarily conserved mechanism by which cytoplasmic proteins and organelles are degraded intracellularly though lysosomal pathway, which has also emerged as a major regulator of cardiac homeostasis and function (Sciarretta et al., 2018). Autophagy exists in three known types thus far, which are macroautophagy, microautophagy, and chaperone-mediated autophagy (CMA). Subcellular behavior of autophagy contains initiation and maturation of autophagosomes as well as fusion of autophagosomes to lysosomes which is regulated mainly by specific autophagy-related (Atg) genes family (Mizushima and Komatsu, 2011; Schinnerling et al., 2015). Normally, autophagy is adaptive to limit derangements and cell death. However, in some conditions, autophagy facilitates cell death, including apoptosis and necrosis (Choi et al., 2013; Sciarretta et al., 2018). Fast-growing studies on cardiomyocyte death and myocardial infarction substantiate this point. Ad-HGF could induce necroptosis and enhance cardiomyocyte proliferation (You et al., 2016). Liu et al. (2016c) demonstrated that it could also remarkably decrease the binding of Bcl-2 to Beclin1 and increase the formation of Beclin1-Vps34-Atg14L complex to promote autophagy in necroptosis process. Additionally, p62 was markedly attenuated rat with Ad-HGF treatment (Figure 2A). S-allyl-cysteine sulfoxide (alliin) treatment in H9c2 cells shows necroptosis inhibition and autophagy promotion by down-regulating RIPK1, RIPK3, and TRAF2 expression meanwhile up-regulating Beclin 1 and microtubule-associated protein 1 light chain 3 (LC3) (Yue et al., 2019) (Figure 2B). Zhang et al. (2020) found Beclin 1 knockdown genetically would impair autophagy flux, which exacerbated oxygen and glucose deprivation (OGD)-induced necroptotic cardiomyocyte death and cardiac dysfunction but would be alleviated by RIPK3 deletion. TNF-α and pan-caspase inhibitor z-VAD-fmk induced RIPK1-RIPK3-mediated necroptosis in H9c2 cells increased the level of LC3-II, an autophagosome-membrane bound form of LC3, and cannot be attenuated by mitochondrial permeability transition pore (mPTP) inhibitors or GSK-3β inhibitors, that hinted at alternative regulation beyond RIPK3-CaMKII-mPTP myocardial necroptosis pathway. TNF-α/zVAD treatment also increased RIPK1-p62 binding notably and reduced p62-LC3 binding which can be inhibited by rapamycin, while Atg5 knockdown can reduce this effect (Goodall et al., 2016; Ogasawara et al., 2017). Mammalian target of rapamycin (mTOR) is a pivotal kinase regulating cell growth and metabolism via different signaling pathways such as PI3K–AKT and MAPK-IKKα/β, etc. (Saxton and Sabatini, 2017; Mossmann et al., 2018). Regulation of autophagy and potential interaction with necroptosis of mTORC1 may also contribute to cardiomyocyte death. Abe et al. (2019) found that mTOC1 inhibition would suppress TNF-α/zVAD-mediated activation of IKKα/β and attenuate RIPK1/RIPK3-mediated necroptosis. In their study, H9c2 cells were treated by TNF-α/zVAD and determined the level of LDH release for necroptosis. mTORC1 inhibitor rapamycin, and mTORC1/2 inhibitor Ku-0063794 could suppress RIPK1-Ser166 phosphorylation and increase RIPK1-Ser320 phosphorylation, a directly MK2 phosphorylation site (Herranz et al., 2015) compared to TAK1 inhibition. Intracellular localization of transcriptional factor EB (TFEB) suggested that rapamycin and necrostatin-1 can induce TFEB nuclear translocation and promote autophagy in a TFEB-dependent manner (Figure 2C). Suppression of TFEB expression abolished the protective effects of mTORC1 inhibitor on both autophagy and necroptosis (Abe et al., 2019).

In addition, RIPK3-mediated necroptosis regulated by CaMKII can conduct in specific processes of myocardial infarction. RIPK3 phosphorylates CaMKII and increases myocardial ROS production which will cause the myocardial infarction (Zhang et al., 2016) (Figure 2D). RIPK3^−/−^ mice undergoing left anterior descending coronary artery ligation developed a myocardial infarction and showed better ejection fraction and less hypertrophy. Inflammation response and ROS activation was restricted by RIPK3 knockout *in vitro* (Luedde et al., 2014). ZYZ-803 is a hybrid molecule of a dual donor for gasotransmitter H2S and NO. Endoplasmic reticulum stress and necroptosis of AMI heart could be attenuated by ZYZ-803 depending on RIPK3-CaMKII signaling pathway regulation (Chang et al., 2019). Evidence suggests that long term xenoestrogen Bisphenol-A exposure would interfere with coronary circulation by activating CaMKII and inducing coronary endothelial necroptosis (Reventun et al., 2020).

### Ischemia-Reperfusion Injury

Reperfusion of ischemic myocardium is a critical strategy for rescuing cardiomyocytes from impendent infarction, whereas it can also cause both reversible and irreversible injury performing as expansion of infarct area and coronary artery dysfunction (Bolli, 1992; Przyklenk, 1997). An indisputable fact has been acknowledged with the continuous cognition of the post-conditioning phenomenon that reperfusion could cause irreversible injury, which can be alleviated by reperfusion modification (Ovize et al., 2010). Apoptosis and necrosis are two crucial mechanisms of early research in ischemia-reperfusion (I/R) injury (Skemiene et al., 2013). Increasing findings unravel that RIPK1/RIPK3-mediated necroptosis also manifest a pivotal effect.

Oerlemans et al. (2012) discovered in 2012 that inhibiting RIPK1 may lead to long-term improvements after ischemia-reperfusion *in vivo*. After left coronary artery (LCA) ligation and 24 h of reperfusion of C57Bl/6 mice, the group treating with RIPK1 inhibitor Nec-1 showed reduction of infarction area and attenuation of inflammatory response. Cardiac remodeling measured by cardiac geometry and fibrosis after I/R for 28 days can be remarkably prevented through RIPK1 inhibition. Similarly, ischemia and reperfusion had been conducted on Guinea pig hearts to verify the effect of RIPK1-mediated necroptosis in myocardial ischemia–reperfusion injury, finding that apoptosis and necroptosis are both involved. These findings indicated that cardio-protection could be promoted by the combination of necroptosis and apoptosis inhibition (Koshinuma et al., 2014). Of note, downregulating of oxidative stress genes CYBA and TXNIP of RIPK1 inhibition provides a novel insight of cardiomyocyte I/R injury and myocardial remodeling (Oerlemans et al., 2012).

Non-classical necroptosis pathway mediated directly by RIPK3 rather than RIPK1 has also been found essential in pathophysiological process of myocardial I/R injury, in addition to RIPK1-RIPK3-MLKL pathway. Zhang et al. (2016) revealed a profound mechanism of RIPK3 in ischemia and oxidative stress–induced myocardial necroptosis. Utilizing RIPK3-deficient (Ripk3^−/−^) mice, they observed that RIPK3 deficiency of mice could block I/R-induced and Dox-induced myocardial necrosis, which did not require the participation of RIPK1 or MLKL. A similar result was also found *in vitro*. Phosphorylation of RIPK3 or oxidation could activate CaMKII and subsequently trigger an opening of the mitochondrial permeability transition pore (mPTP) and myocardial necroptosis. CaMKII as a novel substrate of RIPK3 demonstrated a promising therapeutic strategy of I/R injury (Zhang et al., 2016).

Endoplasmic reticulum (ER) stress can induce cellular death by activating intrinsic mitochondrial apoptosis and RIPK3-mediated necroptosis. RIPK3 was evidently upregulated in I/R injury mice, of which ER stress was induced accompanied by Ca level ([Ca]) and xanthine oxidase (XO) increase and mediated mPTP opening by raising reactive oxygen species (ROS), cardiomyocytes necroptosis occurred ultimately (Saveljeva et al., 2015; Zhu et al., 2018). Melatonin can reverse IR-triggered microvascular perfusion defect and sustain microvascular barrier function via suppressing expression of RIPK3 and prevent endothelial necroptosis by inhibiting the RIPK33-PGAM5-CypD-mPTP cascade (Zhou H. et al., 2018). Through inhibiting RIPK3-MLKL/CaMKII necroptosis pathway, melatonin treatment would attenuate the sensibility of cardiomyocytes to I/R injury induced by chronic pain (Yang et al., 2018).

Recently studies also set sights on mitochondrial function. RIPK3 could intensely perform translocation and expression in mitochondria when mouse model undergoes I/R injury. During cardiomyocyte I/R injury, mitochondria suffered hypoxia/reoxygenation damage resulting in RIPK3-depended mitochondrial fragmentation and necrosis-based death, mediated by increasing lactate dehydrogenase release and inhibiting cell viability. Activation of dynamin-related protein 1 (Drp1) by RIPK3 was found participating in this process, together with the ROS elevation and mitochondrial inner membrane potential (ΔΨm) decline (Hou et al., 2018).

**FIGURE 3:**
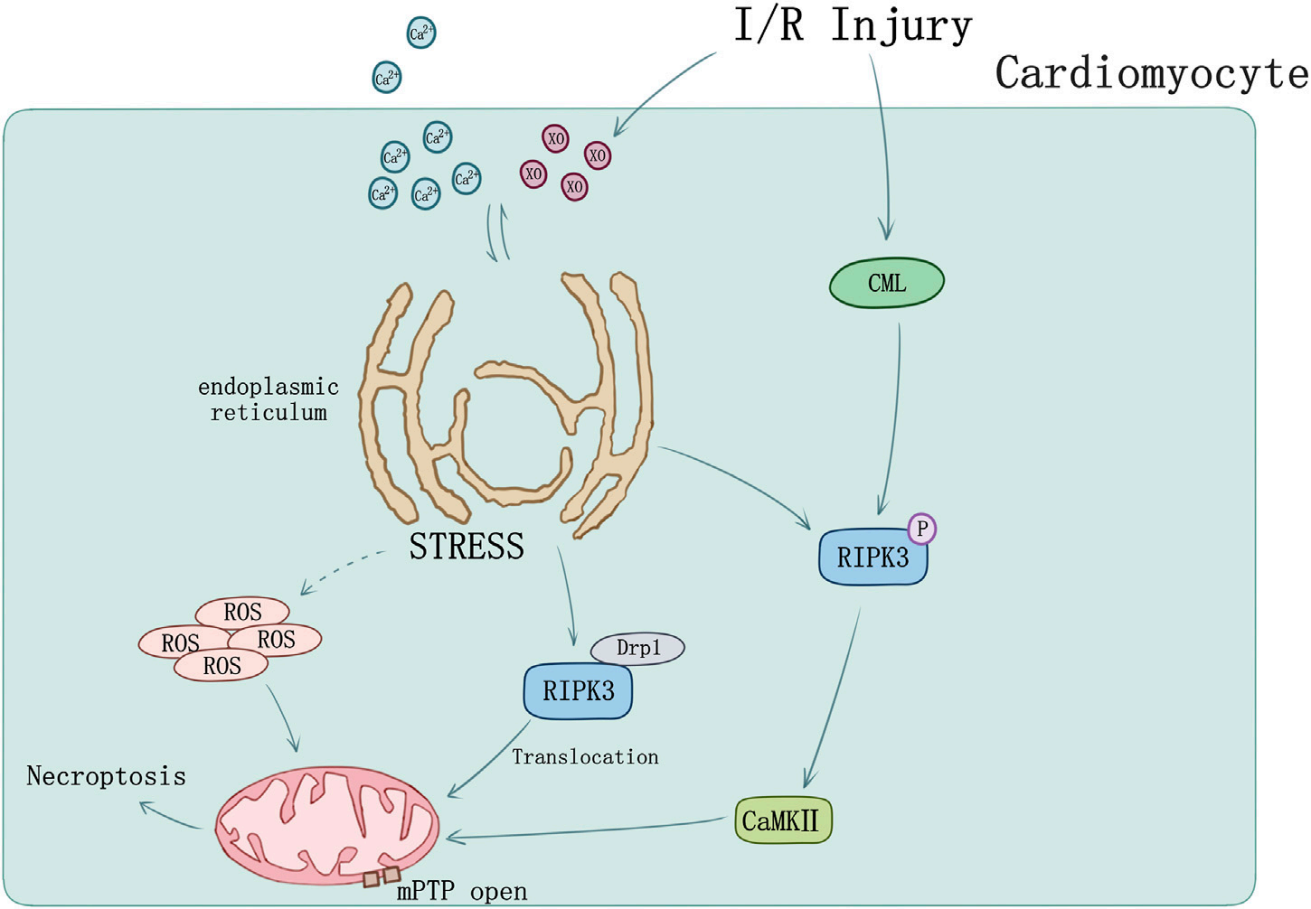
Ischemia-reperfusion injury and ER stress induced myocardial necroptosis. [Ca] and XO influx cause ER stress and mediated mPTP opening by raising ROS. RIPK3 phosphorylation can be induced by CML in I/R injury. Phosphorylation of RIPK3 could activate CaMKII and subsequently trigger opening of the mPTP. I/R could cause RIPK3 and Drp1 translocation from cytoplasm to mitochondrion.

Tumor necrosis factor receptor (TNFR) plays a key role in necroptosis signaling pathway (Dondelinger et al., 2017). TNFR1 knockout *in vitro* blocked the phosphorylation of RIPK3 and decreased APJ, HIF-1α, and VEGF level. Nε-(carboxymethyl) lysine (CML) is the main component of advanced glycation end products. Acute myocardial infarction patients and MI/R mice were both found elevation of CML. RIPK3 phosphorylation can be induced by CML and be blocked by advanced glycation end product (RAGE) receptor knockout as well as glyoxalase-1 overexpression (Yang J. et al., 2019) suggesting novel sight of necroptosis regulation in MI/R injury (Figure 3).

### Cardiac Remodeling

Diversified research recently provided wide sights which contribute to understanding the role of necroptosis in myocardial remodeling. Soluble CD74 receptor ectodomain (sCD74) could perform as a modulator of macrophage migration inhibitory factor (MIF) signaling by diminishing MIF-mediated protein kinase B (AKT) activation and triggering p38 activation. sCD74 could induce necroptosis in cardiac myofibroblasts via inhibiting MIF-mediated survival pathway through C-X-C chemokine receptor 4/AKT axis (Soppert et al., 2018). Autophagy interacts with necroptotic cell death and is also a participant in myocardial remodeling (Liu et al., 2016b; Zhang et al., 2020). High glucose (HG) status may induce inflammation response and then cause cardiac injury and myocardial fibrosis (Miura et al., 2003; You et al., 2016). HG would increase the expression of TLR4 and RIPK3. ATP-sensitive K+ (KATP) channel opener such as diazoxide and pinacidil would blocked the up-regulation of TLR4 and RIP3, suggesting that KATP channel opening can protect myocardium against HG-induced injury and inflammation by inhibiting ROS-TLR4-necroptosis pathway (Liang et al., 2017b). ROS positively interacted with necroptosis demonstrate a novel damage mechanism in HG-induced cardiac injury and inflammation (Liang et al., 2017a). Stimulation of aldehyde dehydrogenases 2 (ALDH2) in high glucose-induced primary cardiomyocytes injury model prevents the occurrence of fibrosis, apoptosis, and necroptosis via inhibiting oxidative stress and inflammation, together with the increased expression of tissue inhibitors of matrix metalloproteinase 4 (TIMP4) protein and the decreased level of matrix metalloproteinase 14 (MMP14) protein level. The mRNA and protein levels of RIPK1, RIPK3, and MLKL decreased (Kang et al., 2020). These provide a new mechanism for myocardial protection.

### Myocarditis

RIPK1/RIPK3-mediated necroptosis regulates inflammatory pathological changing of myocardium. Mouse model of acute viral myocarditis (VMC) induced by Coxsackievirus B3 (CVB3) showed highly expressed of RIPK1/RIPK3, which could be rescued by Nec-1 treatment. Inhibiting the necroptosis pathway may serve as a new therapeutic strategy for acute viral myocarditis (Zhou F. et al., 2018).

*Streptococcus pneumoniae* (the *pneumococcus*) can invade the heart and then cause myocarditis. Pneumococcal myocardial invasion induces inflammation response and immune cell infiltration. Pneumococci releasing pneumolysin via bacterial strain-specific manner kills infiltrated macrophages by activating necroptosis, which alters the immune response (Gilley et al., 2016). The nonhuman primate (NHP) model was used to investigate whether *S. pneumoniae* can cause heart translocation and induce cardiac toxicity. *S. pneumoniae* was detected in the myocardium of all NHPs with acute severe pneumonia. Necroptosis and apoptosis were detected in the myocardium of both acute and convalescent NHPs. It can be treated as an efficient therapeutic target for treatment of severe pneumonia by inhibiting the necroptosis pathway, especially in patients who experience major adverse cardiac events (Reyes et al., 2017).

The interaction and crosstalk of necroptosis and autophagy can change the way of cell death and reduce damage. Myocardial infarction and ischemia-reperfusion injury are related to RIPK3-dependent necroptosis. The upstream molecules related to TNF-induced necroptosis can inhibit or promote myocardial remodeling. Myocarditis is associated with the increase in RIPK1/3.

## RIPK1/3 in Vascular Diseases

### Atherosclerosis

Atherosclerosis is a chronic disease of the vessel wall involving inflammation response of multiple cell types driven by lipid deposition. The process of atherosclerosis would hold a huge reserve of latent period. Once atherosclerotic plaque ruptures, urgent life-threatening cardiovascular events would occur such as myocardial infarction (Kavurma et al., 2017). The basic pathophysiological mechanism of atherosclerosis includes incipient subendothelial retention and accumulation of infiltrated LDL, conducting oxidation and aggregation sequentially, and ultimately triggering chronic inflammatory response and immune reaction (Moore and Tabas, 2011; Hansson et al., 2015). Necroptotic cell death found in the necrotic core within atherosclerotic lesions (Lin et al., 2013) provides the possibility of RIPK1/RIPK3-mediate necroptosis signaling pathway investigation in atherosclerosis.

**FIGURE 4:**
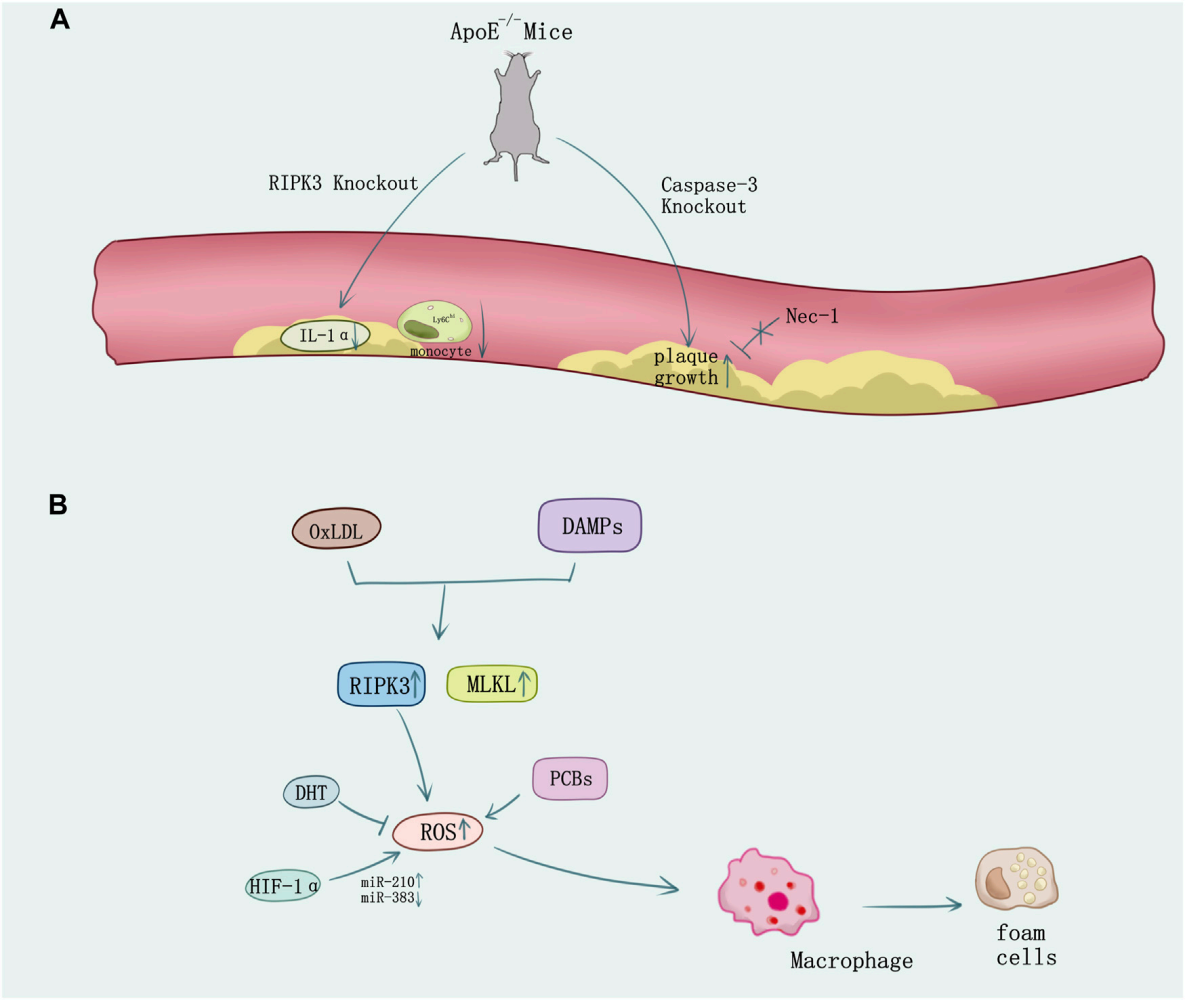
The necroptosis in atherosclerosis. **(A)** In ApoE-knockout-induced spontaneous atherosclerosis mice, knock out RIPK3 causes the decrease of IL-1α and the Ly6C^hi^ monocyte. However, knock out the caspase-3 causes the plaque growth which cannot be reversed by the inhibitor of RIPK1 Nec-1. **(B)** Ox-LDL or DAMPs stimulation released from necrotic cells induces necroptosis in macrophage and the formation of foam cells. ROS overproduction promotes necroptosis which can be suppressed by oxidative stress inhibition using DHT. HIF-1α promotes macrophage necroptosis and ROS production by regulating miR-210 and miR-383.

Macrophages perform major immune and inflammatory functions in atherosclerosis, including recruitment, homing, migration, and differentiation of monocytes, phagocytosis of modified cholesterol, proinflammatory cytokines, enzymes, and ROS secretion (Kavurma et al., 2017). These pathophysiological behaviors not only induce cell death and inflammatory response, but also contribute to foam cell transformation and vulnerable plaque formation (Yahagi et al., 2016). Bao et al. (2006) observed that high levels of sitosterol and other plant sterols would induce premature atherothrombotic vascular disease and macrophage death through autophagy and necroptosis. In ApoE-knockout-induced spontaneous atherosclerosis mice model under RIPK3−/−background, inflammation in atherosclerotic plaques was attenuated and the survival time of atherosclerosis mice got prolonged (Lin et al., 2013). The mRNA expression levels of inflammatory cytokines such as IL-1α, TNF-α, and IL-2 were decreased, lymphocyte infiltrations in the adipocyte tissue and in skin lesions were mitigated, and the high percentage of inflammatory monocytes with high serum levels of lymphocyte antigen 6C (Ly6Chi) which is an inflammatory marker for atherosclerotic risk was greatly decreased in the ApoE/RIPK3 double-knockout mice (Figure 4A). The double-knockout mice presented delayed mortality dramatically in addition (Meng et al., 2015). Thus, antagonism of IL-1α would reduce atherosclerotic lesion mediated by necroptosis (Meng et al., 2016). Macrophage’s necroptosis and genetic expression increase of RIPK3 and MLKL was induced by OxLDL and DAMPS released from necrotic cells driving ROS activation *in vitro*. Mice experiments showed using small-molecule necroptosis inhibitor Nec-1 could reduce atherosclerosis lesions (Karunakaran et al., 2016). Consistent with studies on RIPK3, recently a study indicates MLKL may directly contribute to atherosclerosis lesion development and necrotic core formation. Administration of antisense oligonucleotides down-regulated MLKL expression in ApoE−/− mice alleviated both programmed cell death and necrotic core in the plaque, whereas the total lesion area remained.

Increasing evidence showed diverse methods on regulation of necroptosis which may mediate the atherosclerosis process. Caspase 3 deletion in ApoE−/− mice promoted plaque growth and plaque necrosis but did not sensitize cells to undergo RIPK1-dependent necroptosis (Grootaert et al., 2016) (Figure 4A). However, 5-aminolevulinic acid-mediated sonodynamic therapy would activated the caspase 3 and caspase 8 pathways in foam cells, which is responsible for the switch from necroptosis to apoptosis and may improve the prognosis of atherosclerosis (Tian et al., 2016). Pituitary adenylate cyclase-activating polypeptide (PACAP) plays an important role in cytoprotection, inflammation, and cardiovascular regulation. PACAP/ApoE-deficiency mice showed increasing necroptotic process in atherosclerotic plaques (Leon et al., 2019). Activation of NF-κB in smooth muscle cells (SMCs) is integral to atherosclerosis and involves reversible ubiquitination that activates proteins downstream of pro-atherogenic receptors. TNFR1 activates NF-κB through cascades of polyubiquitination that culminate in the activation of IKK and phosphorylation of IκBα responding to TNF (Peltzer et al., 2016; Jean-Charles et al., 2018), and ubiquitin-specific protease 20 (USP20) would deubiquitinate RIPK1 and alleviate TNF and IL-1β-evoked atherogenic signaling in SMCs accordingly, which add to the evidence demonstrating that SMC-specific gene expression affects atherosclerosis (Subramanian et al., 2010; Liu D. et al., 2016; Jean-Charles et al., 2018). The pathogenesis of atherosclerosis is associated with oxidative stress (Witztum, 1994; Kavurma et al., 2017). Necroptosis plays a vital role in ROS activation (Zhang et al., 2016). Evidence shows that polychlorinated biphenyls (PCBs) promoted the macrophage formation of foam cells, inflammation, and cell necroptosis via ROS overproduction (Yang B. et al., 2019). However, dihydrotanshinone I (DHT) would suppress RIPK3-mediated necroptosis of macrophage to stabilize vulnerable plaque by reducing oxidative stress (Zhao et al., 2021). New findings unveil hypoxia-inducible factor (HIF-1α) upregulates miR-210 and downregulates miR-383 levels in lesioned macrophages and inflammatory bone marrow-derived macrophages. In contrast to miR-210, which inhibited oxidative phosphorylation and enhanced mitochondrial reactive oxygen species production, miR-383 increased ATP levels and inhibited necroptosis by suppressing poly(ADP-ribose) glycohydrolase *(Parg)*, which enhanced atherosclerosis (Karshovska et al., 2020) (Figure 4B).

RIPK3 has been identified to have an atherogenic effect (Lin et al., 2013; Karunakaran et al., 2016), athero-protective function in macrophages and endothelial cells have also been reported lately. RIPK3 may play an anti-inflammatory role by suppressing MCP-1 expression in macrophages and E-selectin expression in endothelial cells, rather than classic function of promoting necroptosis or IL-β processing (Weinlich et al., 2017; Colijn et al., 2020). Novel insights about RIPK3 in atherosclerosis emerge endlessly.

### Abdominal Aortic Aneurysm

Abdominal aortic aneurysm (AAA), characterized by depletion of SMCs, inflammation, negative extracellular matrix remodeling, and progressive expansion of aorta is a major potentially lethal aortic disease with no available pharmacological treatment (Baxter et al., 2008). The pathophysiology of AAA remains incompletely understood. Studies using human specimens and animal models have shown that the infiltrating inflammatory cells, such as macrophages and mast cells, are the major source of both proinflammatory cytokines and matrix-degrading enzymes accumulated in the aneurysmal aortae (Longo et al., 2002; Shimizu et al., 2006). Levels of RIPK3 as well as RIPK1 are elevated in human tissues affected by various pathological conditions, including ischemic stroke, atherosclerosis, and aortic aneurysm. Wang et al. (2015) recently confirmed increased expression of RIPK3 in AAA. In isolated aortic SMCs, knockdown or knockout RIPK3 attenuated TNFα-induced phosphorylation of p65 serine536 and expression of several proinflammatory cytokines. P65 serine536 is critical for enhancing the transcriptional activity of NF-κB and the cytokines regulated by NF-κB such as IL-6, TNF, and VCAM-1 are changed with the activity of NF-κB signaling pathway. Zhou et al. (2019) demonstrate that in AAA, RIPK3 deficiency inhibits aneurysm formation via suppressing cell necrosis and inflammatory response of aortic SMCs. They screened 1141 kinase inhibitors having abilities to block necroptosis and virtual binding to RIPK3, finding GSK074 showed structural similarity to GSK843, a necroptosis blocker in several cell lines such as mouse SMCs. Besides, GSK074 can be bound by both RIPK1 and RIPK3 rather than cause profound apoptosis. Treating with GSK074 can diminish cell death and macrophage infiltration in mouse SMCs and mouse models of AAA histologically.

Studies also unravel RIPK1 plays an extraordinary role in the progression of AAA. Wang et al. (2017) observed amelioration in mouse abdominal aortic aneurysm model by inhibiting RIPK1 with Nec-1. They treated mouse SMCs and elastase-induced murine AAAs with TNFα and zVAD causing necroptosis and then inhibited RIPK1 by Nec-1. The injection of Nec-1 in hypercholesterolemic ApoE−/− mice 30 min ahead of AAA induction showed significantly alleviated aneurysm formation. Interestingly, after treating Nec-1 for another 7 days, at the 8th day of elastase perfusion, smaller mean aortic expansion and marked reduction of macrophage infiltration and MMP9, which was produced primarily by inflammatory cells and plays important roles in aneurysm pathogenesis was observed, suggesting inhibition of RIPK1 can block progression in mice with pre-existing small AAAs.

MLKL expression is also tested in elastase-induced AAA rat model. Within angiotensin AT2 receptor agonist compound 21 (C21) treating, twofold of MLKL expression was decreased as compared with the vehicle group; meanwhile, the expression of other inflammatory mediators such as IL-1β, NF-κB, MMP9, and TGF-β1 were down-regulated likewise (Lange et al., 2018). Histologically, MLKL was found mostly in the media colocalizing with vascular smooth muscle cells and inflammatory cells (Marin et al., 2018). Although changes in genetical and protein levels of MLKL have been verified in AAA, further research remains to better understand the relationship between MLKL and AAA.

Stimulator of interferon genes (STING), a proinflammatory molecule in the cyclic GMP-AMP synthase (cGAS)–STING cytosolic DNA sensor signaling pathway (Ablasser et al., 2013), has previously been shown to promote necroptosis partially through tumor necrosis factor receptor signaling (Sarhan et al., 2019). Novel study shows that DNA damage would activate STING-TBK1-IRF3 pathway and induce necroptosis to promote aortic degeneration, via phosphorylating RIPK3 directly (Luo et al., 2020). Potential multiple pathway crosstalk with RIPK1/3-mediated necroptosis requires confirmation.

RIPK3 knockout decreased the infiltration of pro-inflammation cytokines and the trend of macrophages transforming to foam cells in arthrosclerosis. Inhibition of RIPK1/3 and MLKL are expected to be the therapeutic targets of AAA.

## RIPK1/3 in Cardiovascular Transplants

Organ transplant injury occurs with ischemia and alloimmunity. Lau et al. (2013) first confirmed the involvement of RIPK1/3 in donor organ necroptosis to promote inflammatory and ischemia-reperfusion (I/R) injury in kidney transplant models. Subsequently, extensive evidence showed that the suppression of RIPK1/3-induced necroptosis can provide major protective benefits in the diverse organ transplant model (Kwok et al., 2017; Kanou et al., 2018; Kim et al., 2018).

**FIGURE 5:**
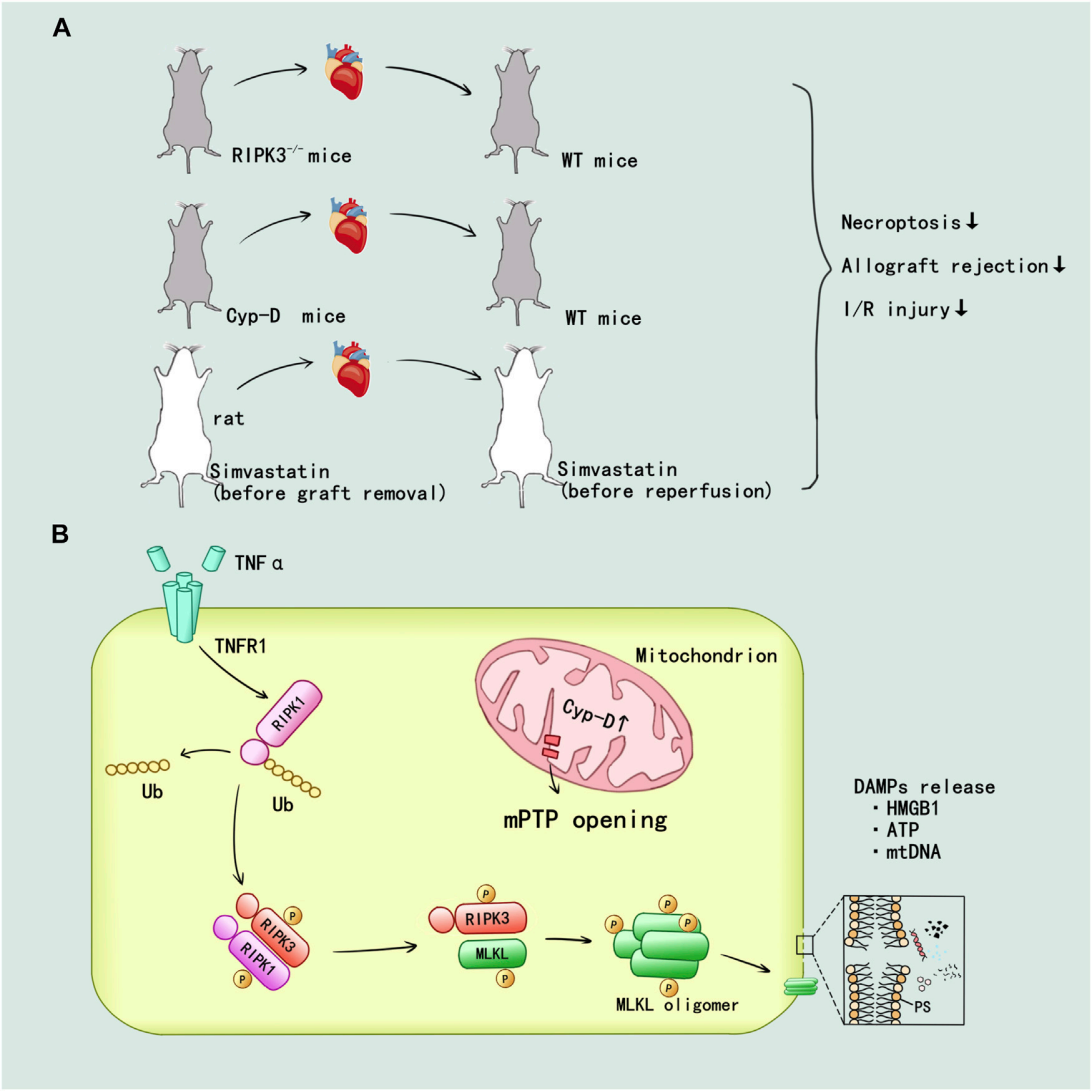
RIPK1/3-mediated necroptosis promoted the inflammatory and I/R injury in heart transplant. **(A)** *In vivo*, the establishment of RIPK3 and Cyp-D knockout have been verified that inhibition of RIPK3 and mitochondrial pathway could alleviate the injury. The simvastatin pretreatment in donor and recipient attenuated the allograft rejection and I/R injury. **(B)** TNF-α can promote the deubiquitination of RIPK1 through TNFR1 mediated intracellular signal transduction. mPTP opening mediated by Cyp-D facilitate the formation of RIPK1/RIPK3/MLKL necrosome and MLKL phosphorylation. Then, MLKL ultimately mediated the cell membrane rupture and released a variety of DAMPs.

Pavlosky et al. (2014) first confirmed the mechanism of RIPK1/3-mediated donor graft rejection in cardiac transplantation: TNFα induced necroptosis in murine cardiac microvascular endothelial cell (MVEC) required expression of TNFR1 and RIPK3. Then RIPK1/3 promoted the necroptotic death and release of the danger molecule HMGB1 in MVEC. The RIPK3−/− mice showed alleviated lymphocyte infiltration and damage in endothelium and myocyte, also RIPK3 deficiency in heart allografts prolongs graft survival after transplantation (Figure 5A). CD4^+^ T cell also could induce MVEC death via released TNFα. The cytotoxicity of CD4^+^ T cell depended on a RIPK3-induced necroptosis in MVEC, and the loss of RIPK3 could reduce chronic cardiac allograft rejection (Kwok et al., 2017) (Figure 5A). In addition, prolonged cold ischemia could significantly enhance expression of RIPK1/3 in cardiac allograft. In addition, the simvastatin pretreatment reduced mRNA expression of RIPK1/3 and protein activity of RIPK1 in cardiac allograft I/R injury as well as prevented necroptotic pathway activation in cardiac allograft (Tuuminen et al., 2016) (Figure 5A).

The mitochondrial permeability regulated by cyclophilin D (Cyp-D) has been confirmed to participate in the RIPK3-mediated necroptosis and rejection in donor heart: Cyp-D promoted the phosphorylation of RIPK3-downstream mixed lineage kinase domain-like protein (MLKL), resulting in cell membrane rupture. Cyp-D–deficiency contributed to prolonged survival in cardiac grafts (Gan et al., 2019) (Figure 5A). Researchers speculated that CypD-mediated mitochondrial permeability transition pore (mPTP) opening might provide enzymes and ATP for the formation of RIPK1/RIPK3/MLKL necrosome and MLKL phosphorylation, but this idea needed to be further verified. The details of RIPK1/3-mediated necroptosis promoting the inflammatory and I/R injury in heart allograft were displayed in Figure 5B.

## Conclusion

Thus far, cardiovascular disease still is the major reason of global death. Although there are many surgical and medical treatments for cardiovascular diseases, there is no treatment through immunotherapy and inhibition of necroptosis. As a key regulator of necroptosis, RIPK1/3 can participate in the occurrence of cell death and inflammation in cardiovascular diseases. In myocardial infarction, ischemia-reperfusion injury, cardiac remodeling, and myocarditis, inhibiting RIPK1/3 would interrupt the transmission of inflammatory signals, thereby reducing the damage and remodeling of cardiomyocytes. Similarly, in atherosclerosis and abdominal aortic aneurysms, inhibiting RIPK1/3 can reduce the formation of foam cells and the development of small aneurysms. In a variety of inflammatory signaling pathways, the RIPK family is very important and is expected to become a key target for therapy.

## Glossary

APJ: angiotensin domain type 1 receptor-associated proteins
ApoE: apolipoprotein E
cIAP: cellular inhibitors of apoptosis
CML: ε-(carboxymethyl) lysine
CypD: cyclophilin D
DAMP: damage-associated molecular pattern
DD: death domain
DR: death receptor
Drp1: dynamin-related protein 1
HGF: hepatocyte growth factor
HIF-1: hypoxia-inducible factor-1
HMGB1: high mobility group protein 1
LC3: microtubule-associated protein 1 light chain 3
LUBAC: linear ubiquitin chain assembly complex
MAPK: mitogen-activated protein kinase
MLKL: mixed lineage kinase domain-like
mPTP: mitochondrial permeability transition pore
NF-κB: nuclear factor κB
OxLDL: oxidized low-density lipoprotein
RHIM: RIP homotypic interaction motif
RIPK1/3: receptor interacting protein kinases 1/3
ROS: reactive oxygen species
TAB: TAK1-binding proteins
TAK1: transforming growth factor-b-activated kinase 1
TFEB: transcriptional factor EB
TLR: toll like receptors
TNF: tumor necrosis factor
TNFR: tumor necrosis factor receptor
TRAF: TNFR-associated factors
TRAIL: TNF-related apoptosis-inducing ligand
ZBP-1: Z-DNA-binding protein 1
